# Low stroke incidence in the TEMPiS telestroke network during COVID-19 pandemic: Effect of lockdown on thrombolysis and thrombectomy

**DOI:** 10.1177/1357633X20943327

**Published:** 2022-08

**Authors:** Felix Schlachetzki, Carmen Theek, Nikolai D Hubert, Mustafa Kilic, Roman L Haberl, Ralf A Linker, Gordian J Hubert

**Affiliations:** 1 Department of Neurology, University of Regensburg, Bezirksklinikum Regensburg, TEMPiS Telemedical Stroke Center, Regensburg, Germany; 2 CTS, Herdecke, Germany; 3 Department of Neurology, TEMPiS Telemedical Stroke Center, Academic Teaching Hospital of the University of Munich, München Klinik Harlaching, Munich, Germany

**Keywords:** Telestroke, COVID-19, lockdown, stroke, thrombolysis, telehealth

## Abstract

**Background:**

During the COVID-19 pandemic emergency departments have noted a significant decrease in stroke patients. We performed a timely analysis of the Bavarian telestroke TEMPiS “working diagnosis” database.

**Methods:**

Twelve hospitals from the TEMPiS network were selected. Data collected for January through April in years 2017 through 2020 were extracted and analyzed for presumed and definite ischemic stroke (IS), amongst other disorders. In addition, recommendations for intravenous thrombolysis (rtPA) and endovascular thrombectomy (EVT) were noted and mobility data of the region analyzed. If statistically valid, group-comparison was tested with Fisher’s exact test considering unpaired observations and ap-value < 0.05 was considered significant.

**Results:**

Upon lockdown in mid-March 2020, we observed a significant reduction in recommendations for rtPA compared to the preceding three years (14.7% [2017–2019] vs. 9.2% [2020], p = 0.0232). Recommendations for EVT were significantly higher in January to mid-March 2020 compared to 2017–2019 (5.4% [2017–2019] vs. 9.3% [2020], p = 0.0013) reflecting its increasing importance. Following the COVID-19 lockdown mid-March 2020 the number of EVT decreased back to levels in 2017–2019 (7.4% [2017–2019] vs. 7.6% [2020], p = 0.1719). Absolute numbers of IS decreased in parallel to mobility data.

**Conclusions:**

The reduced stroke incidence during the COVID-19 pandemic may in part be explained by patient avoidance to seek emergency stroke care and may have an association to population mobility. Increasing mobility may induce a rebound effect and may conflict with a potential second COVID-19 wave. Telemedical networks may be ideal databases to study such effects in near-real time.

## Introduction

Implementation of social distancing to combat the impact of Corona virus pandemic sequelae has emerged as the major strategy to contain the spread of infection given the lack of specific treatments for COVID-19 and limited intensive care resources.^1^ Major concerns for stroke neurologists in this extraordinary scenario include the following: (a) rapid specific management of cases of acute stroke with possible COVID-19 from initiation of the stroke call in the preclinical setting through the ambulance system, emergency department, and hospital stroke department, and in the neuroradiological department when needed to aid in stroke diagnosis and treatment; (b) the fact that patients with mild stroke symptoms or transient ischemic attacks (TIAs) may be reluctant to request hospital admission for acute stroke;^2^,^3^ and (c) that COVID-19 itself is associated with severe stroke syndromes. This is suggested in a recent case series of COVID-19 patients from Wuhan, China, focusing on neurological symptoms, that described cerebrovascular events in 6 of 214 cases (6.3%), especially in elderly patients and in those with more severe infections. Also, authors of a second case series reported unusual cases of young COVID-19 patients (<50 yrs) with large vessel stroke; and other authors reported three stroke patients with coagulopathy and antiphospholipid antibodies in the context of severe COVID infections.^4–6^

In contrast, several stroke departments in Germany (including our own), the USA and China have noted a significant drop in the number of stroke patient admissions during the Corona pandemic.^7^ Data on this phenomenon are still scarce; however, in a descriptive report by Morelli et al. from Piacenza, Lombardy, Italy, covering the period 21 February (appearance of the first SARS-CoV-2 patient recorded in Italy) to 25 March 2020, the number of stroke admissions decreased from an average of 51 (with 21% large vessel occlusions (LVOs)) to 6 (two TIAs, one LVO and three lacunar strokes).^8^ Using a commercial neuroimaging database with the RAPID software platform, Kasangra and Hamilton observed a 39% decrease in stroke imaging procedures with the nadir following the first statewide stay-at-home order in the USA.^9^ The decrease was observed in all age, sex, and stroke severity subgroups within all 856 participating hospitals, which processed overall 213,573 patients between 1 July 2019 and 27 April 2020. Cardiologists in France observed a similar significant drop in admissions to nine intensive cardiac care units after initiation of social distancing and self-quarantine in mid-March 2020.^10^ Overall, there are scarce data available on the impact of the COVID-19 infection itself on cardiovascular morbidity including cerebral stroke.^11^

## Aims and hypothesis

The primary aim of this study was to evaluate the effect of the COVID-19 pandemic lockdown on stroke consultations and treatment recommendations using the acute consultant database of the telestroke network TEMPiS.^12^ We focused on data collected during the first four months of 2020, which included the emergence of the Corona virus pandemic in Southeastern Bavaria through the first two months of social distancing/region shutdown. We compared these data with comparable data collected during the same months in the years 2017–2019.

## Methods

Data from daily consultations at 12 clinics without neurology departments in the telestroke network TEMPiS form the basis of this study. The consultations took place between 1 January and 30 April in the years 2017–2020. All data were pseudonymized. We extracted the actual working diagnoses based on telemedical consultation and neuroimaging results, mainly cerebral computed tomography. Two major databases were used to calculate the population within these districts (www.destatis.de and https://experience.arcgis.com/experience/478220a4c454480e823b17327b2bf1d4/page/page_1/). This retrospective study was approved by the local ethics committee of the University of Regensburg (20-1789-104) and performed in accordance with guidelines of the Declaration of Helsinki. Mobility data available at https://www.apple.com/covid19/mobility were extracted; these data were generated from the relative request volume for directions in Munich, Germany compared with a base volume on 13 January 2020. To observe the relationship of mobility and the reported stroke decline in Piacenza we also extracted mobility data from Milan, close to Piacenza, Italy.^8^

The major ‘working diagnostic groups’ were as follows: (a) ischemic stroke; (b) TIA; (c) intracranial haemorrhage; (d) epileptic seizure; (e) migraine; and (f) other disorder (including facial palsy, headache and brain tumour). Also included were cases in which there were recommendations for IV thrombolysis (IV rtPA) or endovascular therapy (EVT, thrombectomy) for LVO.

Exploratory descriptive summary statistics with mean values and standard deviations were applied in an analysis of data covering January through April in years 2017–2019 in comparison with data covering the same period in 2020. Counts are presented as a graphic display showing incidences standardized to 15-day periods. If statistically valid (especially percentage of recommendations for IV thrombolysis and thrombectomy) group-comparison was tested with Fisher’s exact test considering unpaired observations. A *p*-value <0.05 was considered significant.

## Results

**Figure 1.**
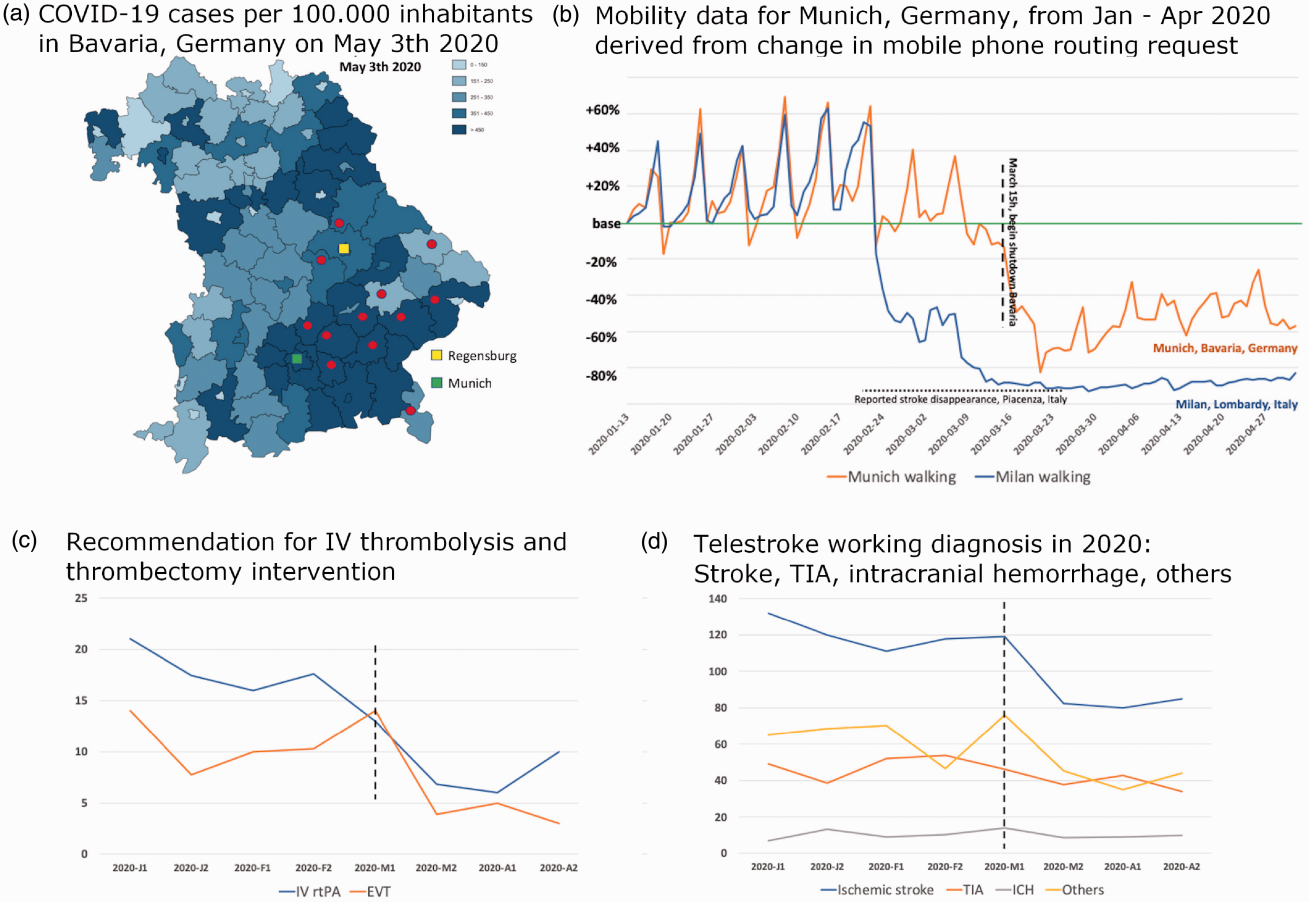
(a) Incidence of new COVID-19 infections in Bavaria on April 18, 2020. Red dots indicate network hospitals, and green and yellow squares depict the two academic stroke centres that alternate weekly for the TEMPiS consult service. Modified with permission from the Bavarian State Office for Health and Food Safety. http://www.lgl.bayern.de/gesundheit/infektionsschutz/infektionskrankheiten_a_z/coronavirus/karte_coronavirus/; (b) Mobility data according to COVID-19 - Mobility Trends Reports - Apple. The data reflect requests for routing in Apple maps for Munich, which resides in the centre of the TEMPiS network, and for Milan near Piacenza, where the first decline in the number of strokes was reported (Morelli et al.^8^). Horizontal dotted line indicates reported reduced stroke activity in Piacenza. (c) Recommendations (absolute numbers) for application of IV thrombolysis and thrombectomy. Vertical dashed line indicates the official beginning of lockdown in Bavaria. Time and patient numbers on y-axis are standardized to 15-day periods (x-axis) in each month to compensate for shorter (February) and longer (January and March) months. 2020 J1 = January first half, 2020 J2 – January second half; F = February, M = March, A = April. (d) Working diagnoses of the telestroke consultations. Vertical dashed line indicates the official beginning of lockdown in Bavaria. Time and patient numbers on y-axis are standardized to 15-day periods (x-axis) in each month to compensate for shorter (February) and longer (January and March) months. 2020 J1 = January first half, 2020 J2 – January second half; F = February, M = March, A = April.

There were 7637 telemedical consultations during the specific time frames investigated, and the population in the geographical areas covered by these 12 rural hospitals is 1,273,000. Most hospitals reside in areas with a high number of COVID-19 cases (Figure 1(a)). The number of COVID-19–positive cases in the whole of Bavaria rose from five at the end of February 2020 to 42,782 cases on 30 April 2020. The public lockdown was initiated on 15 March 2020; however, the recommendation of personal quarantine for people who had travelled to Northern Italy was broadcast earlier, on 9 March. In Munich, Apple® mobility trends demonstrated a decrease in walking activity in mid-March 2020 to –60% (–40 to –80%) of the baseline level. In Milan, Lombardy, Italy, on 25 March walking activity began to decrease, soon reaching –80% of baseline activity and remaining fairly constant thereafter (Figure 1(b)).

Overall, 7608 consultations were analysed and 29 excluded being non-acute consultations within the network (i.e. follow-up examinations). Statistically significant changes in the number of recommendations for IV thrombolysis were observed in 2020 (Figure 1(c)). While in 2017–2019 IV thrombolysis was recommended in 14.7% of consultations with suspected ischemic stroke (148 of 1006), the frequency of this recommendation decreased to 9.2% (23 of 250) in 2020 (*p* = 0.0232). No differences in the number of IV thrombolysis recommendations were observed during the time period covering 1 January to 15 March (13.8% in 2017–2019 vs. 14.2% in 2020; not significant). No trend in fewer recommendations for EVT was observed between 16 March and 30 April in 2020 compared with the same time periods in 2017–2019 (2020: 7.6% (19 of 250) vs. 2017–2019 7.4% (74 of 1006)). However, in the preceding time frame 1 January to 15 March 2020, significantly more recommendations for thrombectomy were made compared with 2017–2019 (2020: 9.3% (56 of 600) vs. 5.4% (88 of 1619) in 2017–2019; *p* = 0.0013).

The data reflect the development of consultations and treatment recommendations for LVO in the network from 2017 onward. The number of recommendations for EVT steadily rose, with increasing evidence for recanalization even in later time windows and increasing employment of computed tomography angiography in the TEMPiS network. Table 1 shows the development of consultations in 2020 up to the end of the study including the lockdown period; it shows a drop in the number of consultations and, more importantly, fewer recommendations for IV thrombolysis and EVT, which suggests fewer incidences of ischemic stroke severities (Table 1, Figure 1(d)).

**Table 1.** Total number of consultations for January–April 2020, divided into 2-week periods but without adjustments for different lengths of the month. Data in square brackets show data from 2017–2019 in mean values and standard deviation.

Teleconsultation Stroke Network TEMPIS 2020									
	01–15 Jan 2020	16–31 Jan 2020	01–15 Feb 2020	16–29 Feb 2020	01–15 Mar 2020	16–31 Mar 2020	01–15 Apr 2020	16–30 Apr 2020	
Total COVID-pos. cases in Bavaria	**0**	**0**	**0**	**5**	**1231**	**18,061**	**35,808**	**42,782**	
Consultations2017–2019 mean ± SD	**270** [230 ± 12]	**263** [257 ± 18]	**256** [237 ± 17]	**237** [213 ± 5]	**276** [246 ± 19]	**197** [263 ± 39]	**179** [232 ± 17]	**197** [225 ± 8]	
Ischemic stroke2017–2019 mean ± SD	**132** (49%) [95 ± 12]	**124** (47%)[122 ± 9]	**111 (43%)** [104 ± 5]	**114** (48%) [99 ± 6]	**119** (43%) [120 ± 8]	**85** (43%) [118 ± 25]	**80** (45%) [106 ± 19]	**85** (43%) [111 ± 10]	
IV thrombolysis2017–2019 mean ± SD	**21** (16%) [9.7 ± 1.5]	**18** (15%) [18.4 ± 1.7]	**16** (14%) [15.3 ± 2.9]	**17** (15%) [13.6 ± 6]	**13** (11%)**^†^** [18 ± 2]	**7** (8%) [16.4 ± 5.4]	**6** (8%) [17.6 ± 2.1]	**10** (5%)**^‡^** [14.7 ± 2.1]	**^†^*p* = 0.7912 n.s. ^‡^*p* = 0.0234**
Thrombectomy2017–2019mean ± SD	**14** (11%) [4 ± 2.6]	**8** (6%) [8.1 ± 3.1]	**10** (9%) [6 ± 1.7]	**10** (9%) [5 ± 2.7]	**14** (12%)**^§^** [6.3 ± 1.2]	**4** (5%) [9.3 ± 5.7]	**5** (6%) [7.7 ± 0.6]	**10** (5%)**^§§^** [7.3 ± 4.7]	**^§^*p* = 0.0016^§§^*p* = 0.1719 n.s.**
TIA2017–2019 mean ± SD	**49** (18%) [43 ± 7]	**40** (15%) [48 ± 8]	**52** (20%) [47 ± 7]	**52** (22%) [37 ± 5]	**46** (17%) [40 ± 5]	**39** (20%) [47 ± 8]	**43** (24%) [48 ± 8]	**39** (20%) [46 ± 2]	
ICH2017–2019 mean ± SD	**7** (3%) [5.7 ± 1.5]	**14** (6%) [9.3 ± 0.6]	**9** (4%) [10.3 ± 4]	**10** (4%) [11 ± 5.2]	**14** (5%) [10.3 ± 2.4]	**9** (5%) [14.3 ± 1.5]	**9** (5%) [7.7 ± 5.1]	**9** (5%) [10 ± 4.6]	
ICB	7	6	7	6	7	6	**6**	5	
SAH	0	6	1	4	5	1	1	2	
SDH	0	2	1	0	2	2	2	3	
Seizure	**8** (3%)	**11** (4%)	**13** (5%)	**11** (5%)	**11** (4%)	**15** (7%)	**10** (5%)	**15** (8%)	
Migraine	**9** (3%)	**3** (1%)	**1** (1%)	**5** (2%)	**9** (3%)	**2** (1%)	**2** (1%)	**2** (1%)	
Others2017–2019 mean ± SD	**65** (24%) [61.3 ± 15.6]	**71** (27%) [63 ± 5.6]	**70** (27%) [61.3 ± 18.1]	**45** (19%) [50.7 ± 11]	**76** (28%) [65.7 ± 10.8]	**47** (24%) [70.3 ± 19]	**35** (20%) [52.7 ± 11.7]	**47** (24%) [39.3 ± 7.4]	

ICB: intracranial bleeding; ICH: intracranial haemorrhage; SAH: subarachnoid hemorrhage; SDH: subdural hematoma; TIA: transient ischemic attacks. Bold numbers are only bold for better readability.

**^†^***p* = 0.7912 n.s.

**^‡^***p* = 0.0234

**§***p* = 0.0016

**§§***p* = 0.1719 n.s.

Although Bavaria is the state with the highest number of COVID-19 cases in Germany, especially in our region, we only performed five telestroke consultations for the 12 network hospitals in which possible COVID-19 infection was discussed (including a single patient with stroke symptoms and fever).

## Discussion

The TEMPiS telestroke working data confirm the current observation of a low stroke incidence in Southeastern Bavaria, with relative proportions of the working diagnosis remaining similar. The number of cases of disabling stroke from intracranial haemorrhage and ischemic stroke requiring IV rtPA or EVT also diminished, challenging the theory that only patient avoidance to call for emergency treatment is responsible for this phenomenon. This study also demonstrates the potential and importance of telestroke networks in the current COVID-19 pandemic.^3^,^13^

The observation of fewer stroke cases during the COVID-19 pandemic seems to contradict two essential assumptions with regard to stroke risk: (a) SAR-COV-2 is a strong risk factor for stroke; and (b) physical inactivity in a lockdown setting may increase the risk of stroke, especially among elderly persons. First, SAR-COV-2 may induce hypercoagulability and high levels of C-reactive protein, D-dimer and interleukin-6, placing patients at risk to develop thrombotic complications.^14^ In a series of 184 intensive care unit patients in the Netherlands, reported by Klok et al., only three strokes complicated the course of COVID-19, whereas the majority of complications included pulmonary embolism (*n* = 25) and peripheral venous thrombosis and catheter-associated thrombosis (*n* = 3).^15^ Observations in case series that concurrent COVID-19 infection complicates or triggers unusual ischemic stroke may well prevail, but case control studies focusing on this phenomenon are urgently needed to affirm or deny the assertion.^5^ Second, physical inactivity has a profound effect on atrial fibrillation, obesity, diabetes mellitus management and hypertension, among others, and contradicts current recommendations on mid- and long-term stroke prevention.^16^ A recent study in 97 consecutive patients with non-ST-segment elevation acute coronary syndromes (ACSs) and optical coherence tomography of the culprit lesion, reported by Kato et al., found that the combination of greater physical activity, outdoor ACS onset, and high body mass index had a significant effect on the incidence of coronary plaque erosion.^17^ Interestingly, mobility data, such as those provided by the Apple mobility database®, demonstrated a parallel reduction in incidences of stroke and ACS in three published papers^8–10^ in addition to ours.

Our data confirm the observation from Morelli et al., who termed the phrase ‘baffling case of ischemic stroke disappearance’.^8^ These authors also discuss that this effect cannot be totally explained merely by the reluctance of patients to call for help in a stroke emergency because the number of cases presenting with severe stroke requiring EVT and the number of general consultations in TEMPiS also decreased. An analysis based on a large database associated with the application of RAPID software in acute stroke by Kansagra et al. is in line with our observation that also severe stroke patients diminished during the early lockdown phase.^9^ The number of ischemic core volumes 100–150 ml and greater than 150 ml were observed to decrease by 39.2% and 45.5%, respectively; core volumes 15–100 ml decreased by 16.6% and 25%; and very small core infarct volumes measuring 0–15 ml decreased 41%.^9^ The decrease in the number of very small infarct volumes may well be explained by the generally proposed hesitation to seek emergency care, while the reduction in large ischemic core volumes is more likely due to fewer LVOs, as observed in our study with a sharp decline in IV thrombolysis and thrombectomy recommendations.

Another explanation may be a concurrent low infection rate with other viruses that can trigger atherosclerosis and plaque rupture resulting in neuro- and cardiovascular morbidity.^18^ The lockdown not only reduces physical activity; strict social distancing and use of facial masks should also lead to low rates of exposure to and transmission of other common viruses and allergens that by themselves appear to trigger stroke.^19^ Additional studies with detailed analyses of symptom onset-to-door times, stroke severity, neuroimaging and inflammatory markers are needed to understand the reason for the reduced number of revascularization therapies requested during the COVID-19 pandemic.

## Limitations of the study

Analysis of daily working diagnoses in the TEMPiS telestroke network has the advantage of being highly timely, yet it lacks specificity because the final diagnosis may differ from the initial one. This may be compensated by the creation of a large common database for telestroke networks that incorporates corrections for the actual population covered, analyses of other stroke-related databases such as the one associated with RAPID software, healthcare provider databases and common stroke registries for quality control. The decrease in the number of thrombectomy recommendations in our cohort mid-March 2020 did not reach statistical significance when compared with the same period in years 2017 through 2019, because rates for this procedure increased according with levels of evidence.^20^,^21^ In agreement with this development, thrombectomy recommendations by TEMPiS neurologists in 2020 prior to the COVID-19 pandemic occurred more frequently than in previous years.

## Conclusions

Our study using the TEMPiS telestroke database confirms lower incidences of ischemic stroke and other acute neurological disorders requiring consultation, such as intracerebral haemorrhage, seizure disorder and migraine. Next to a reluctance within the population to seek immediate medical assistance for acute stroke, the COVID-19 lockdown, which resulted in less physical activity and fewer other common infections, may also be responsible for the fewer number of patients with severe stroke, especially those with intracranial haemorrhage and those eligible for recanalization therapies. If lockdown-associated factors are indeed responsible for a lower stroke incidence, we may expect a rebound effect following the lockdown period, with an increased incidence of stroke (as well as of myocardial infarcts and traumatic brain injuries), as patients’ frailty may have increased during the lockdown. Analyses of large stroke databases may reveal further insights into this phenomenon. However, telestroke networks such as TEMPiS may be ideal tools to monitor stroke occurrence in real time.
